# Clinical development of insulin-like growth factor receptor—1 (IGF-1R) inhibitors: At the crossroad?

**DOI:** 10.1007/s10637-012-9811-0

**Published:** 2012-03-14

**Authors:** Andrea Gombos, Otto Metzger-Filho, Lissandra Dal Lago, Ahmad Awada-Hussein

**Affiliations:** 1 Medical Oncology Clinic, Institut Jules Bordet, 121 Boulevard de Waterloo, 1000 Brussels, Belgium; 2 Université Libre de Bruxelles, Brussels, Belgium

**Keywords:** IGF-1R inhibitors, Monoclonal antibodies, Tyrosine kinase inhibitors, Predictive biomarker

## Abstract

Insulin like growth factor receptor (IGF-1R) targeting became one of the most investigated areas in anticancer drug development during the last decade. Strategies aiming to block IGF-1R activity include monoclonal antibodies, tyrosine kinase inhibitors and anti-ligands antibodies. Initial enthusiasm quickly encountered challenges. Unfortunately the validation of the efficacy of IGF-1R targeted agents in large clinical trials failed, however anecdotal single agent activity was seen in early studies. Consequently, questions regarding the selection of right target population and the appropriate trial design are arising. Despite the plethora of clinical trials conducted no predictive biomarker has been validated so far and resistance mechanisms to IGF-1R inhibitors remain unclear. The other issue to be addressed is how to best combine IGF-1R inhibitors with other therapeutic approaches. This review highlights the most relevant clinical data emphasizing the main tumor types where IGF-1R inhibition showed potential interest. We also tried to extract based on clinical and translational data some candidate biomarkers that could help better to select patient population who potentially could benefit most from this therapeutic approach.

## Introduction

Insulin like growth factor receptor—1 (IGF-1R) is a transmembrane receptor with tyrosine kinase activity found to be over-expressed in many tumor types [1].

The crucial role of IGF-1R receptor signaling in malignant transformation and in tumor cell proliferation and survival makes it a very attractive therapeutic target [2–7]. Furthermore, in preclinical models, IGF-1R signaling demonstrated to interfere with numerous receptor pathways and was implicated in the mechanism to render tumor cell resistant to chemotherapy, antihormonal therapy and to anti-EGFR and HER2 targeted therapies [8–21]. Similarly, mammalian target of rapamycin (m-TOR) inhibitors can activate PI3K-Akt pathway via loss of negative feedback on IRS-1 (insulin receptor substrate—1), an effect that can be suppressed by IGF-1R blockade [22–24].

Recently, the anti-neoplastic activity of IGF-1R antibodies became one of the most investigated in clinical oncology. Almost 30 candidate drugs were tested in more than 70 clinical trials conducted in a wide variety of cancers through academia, pharmaceutical and biotechnology companies. Early clinical data suggested the activity of IGF-1R target-drugs in selected tumor types, such as Ewing’s sarcoma, non-small cell lung cancer, adrenocortical carcinoma, but the initial enthusiasm quickly encountered several challenges and disappointment.

In his review we focus on the most relevant clinical data concerning tumor types where IGR-1R targeting was considered of potential interest. Meanwhile, based on existing clinical and translational data this article describes some potential biomarkers that could help to better identify the patient population who would benefit most.

## IGF-1R pathway

**Fig. 1:**
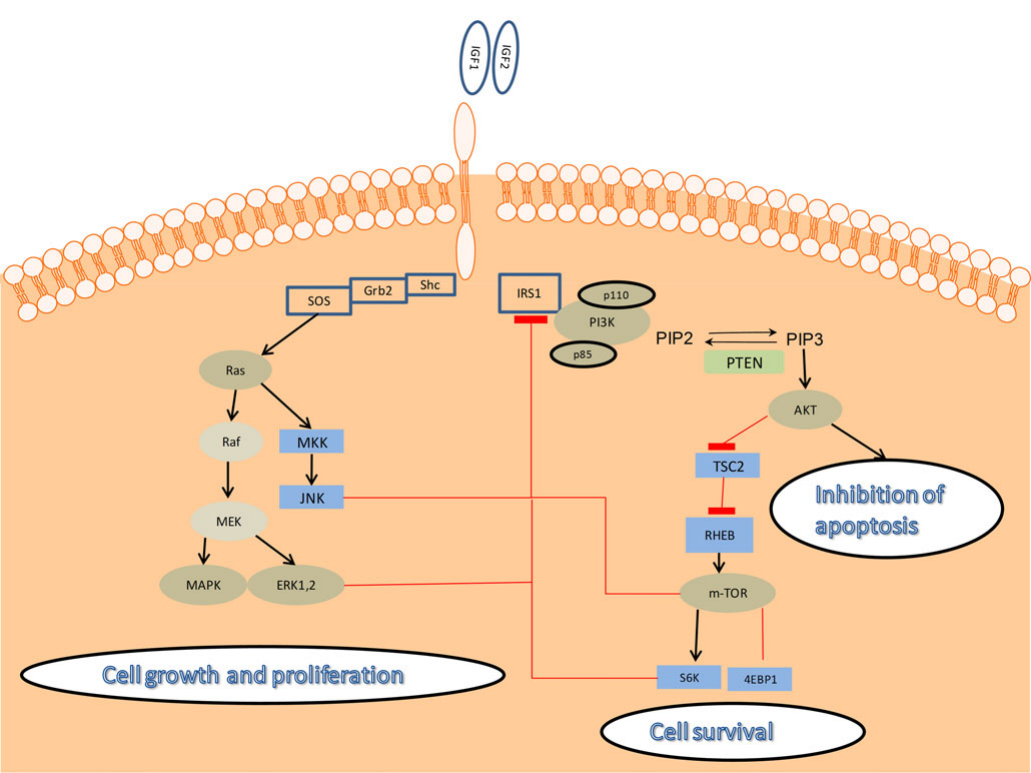
Downstream signaling of the IGF-1R. IGF-1R is a transmembrane tyrosine-kinase receptor, binding either IGF1 or IGF2. Ligand binding leads to IRS phosphorylation and recruitment of regulatory (p85) and catalytic (p110) subunits of PI3K and subsequently Akt phosphorylation on threonine 308. Serine 473 of Akt is phohoshorylated by mTORC2 complex also activated by IGF-1R by an unknown mechanism. Akt promotes cell survival by multiple mechanisms, inhibiting apoptosis and inducing expression of prosurvival genes. The other parallel pathway, related to IGF-1R by IRS or Shc proteins is the RAS-RAF-MAPK and JNK, resulting in cell proliferation. Negative regulation by mTORC1, S6K, JNK and ERKs induces IRS1 degradation. ( AKT—protein kinase ; ERK—extracellular-signal-regulated kinase; MAPK—mitogen activated protein kinase; Grb2—growth factor receptor-bound protein 2; IGF-1R—insulin-like growth factor 1 receptor; GSK3—glycogen synthase kinase 3; IRS 1—insulin-like receptror substrate 1; MEK—mitogen-activated protein kinase; mTOR—mammalian target of rapamycin, PI3K—phosphatidylinositol 3-kinase; PIP2—phosphatidylinositol 4,5-biphosphate; PIP3—phosphatidylinositol 3,4,5 triphosphate; PTEN—phosphatase and tensin homolog; RHEB—Ras homolog enriched in brain; S6K—S6 kinase; SOS—son of sevenless; TSC2—tuberous sclerosis complex 2)

Binding of IGF-1 or IGF-2 to IGF-1R leads to receptor auto-phosphorylation. Ligand bioavailability is elevated in some tissues and is highly dependent on IGF-binding proteins (IGFBP) and IGFBP-proteases. Activation of the receptor leads to the recruitment of multiple adaptor proteins such as insulin receptor substrates (IRS), Shc and Src homologue and subsequent activation of at least two pro-survival signaling pathways. The main event following IGF-1R phosphorylation is the stimulation of phophoinositol 3-kinase (PI3K)-Akt signaling pathway, leading to cell survival. The second pathway consists of Ras, Raf and extracellular-signal-regulated kinase (ERK)/mitogen-activated protein kinase (MAPK) activation, leading to tumor growth and proliferation [4–6]. Figure 1

Although IGF-1R axis components can be highly altered in cancer, little is known about molecular mechanisms involved in this process. Chromosome 15q26, where IGF-1R is located was found to be amplified in basal-like breast cancer [25]. Low levels of IGF-1R copy number gain were also shown in lung cancer [26, 27], pancreatic adenocarcinoma and colon cancer [28–30]. A more recent study has demonstrated that KIT and PDGFR-α wild type GIST have a significantly higher level of IGF-1R amplification than mutated ones [31]. Whereas no mutation of IGF-1R was described to date, there are some reports of gene polymorphism encoding IGF-1 or IGFBP-3.

Several approaches targeting IGF-1R were developed, including monoclonal antibodies, small molecule tyrosine kinase inhibitors and ligand binding antibodies. The most advanced in clinical development are monoclonal antibodies. This approach is considered more selective in blocking IGF-1R activity than tyrosine kinase inhibition. IGF-1R shows high homology with insulin receptor (IR), thus small molecule TKIs may block IR and receptor hybrids as well [2]. This could be an advantage in term of metabolic consequences, considering the high homology between IGF-1R and IR. Selectivity is also important when we take into account treatment efficacy. IR and IR/IGF-1R hybrids represent an important role in IGF signaling, and non-selective TK inhibitors can additionally abrogate their activity. Thus the development of techniques able to differentially assess IGF-1R/IR hybrids and homodimers is necessary to efficiently tailor anti-IGF-1R therapies.

Toxicities reported in phase I trails were uncommon and maximal tolerated dose was not identified for either of the drugs blocking IGF-1R investigated as single agent. Hyperglycemia was more frequently observed with tyrosine kinase inhibitors. Up to 20 % of the patients experienced this toxicity and it was usually mild to moderate and manageable with oral anti-diabetic medication. Other frequently registered toxicities were fatigue (8–14 %), and mild skin toxicities (rash, urticaria and pruritus). Hypersensitivity reaction was a rare event [42, 44, 51, 52]. Hematological side effect was a rare but important when occurred. Grade 3 thrombocytopenia was considered a DLT with 20 mg/kg of AMG-479 and lymphocyte count decrease occurred in 7 % of the patients treated with CP-751, 871 [44, 51].

## IGF-1R inhibitors in lung cancer

Insulin growth factor receptor is overexpressed in up to 80 % of lung cancers (1). Pre-clinical data on cell lines demonstrate that IGF-1R activation induces resistance to anti-epidermal growth factor receptor (EGFR) therapy through activation of PI3K-AKT pathway [12–17].

The first retrospective analysis of IGF-1R expression in primary resected NSCLC (*n* = 184), demonstrated a positive correlation between IGF-1R expression and poor overall survival [32]. The same authors recently concluded that IGF-1R protein expression is significantly higher in squamous cell carcinomas (67 %) when compared with other histologies (24 %). The prognostic value of IGF-1R expression is controversial across different studies [26, 32–34]. As an example IGF-1R gene copy number gain (3 % amplification and 24 % high polysomy) had surprisingly positive prognostic value [26]. High expression level of IGF-1R was related to treatment response in a preclinical study on 22 NSCLC cell lines [26] and in a small cohort of patients treated with figitumomab [26, 27, 35]. The predictive value of IGF-1R copy number gain needs further investigation.

The initial hints of anti-IGF-1R activity in advanced NSCLC observed across several phase I studies were subsequently reproduced in one randomized phase II trial [36]. These trials investigated the efficacy of figitumomab, a selective fully human IgG2 monoclonal antibody against insulin like growth factor receptor 1. A total of 156 patients were allocated to carboplatin, paclitaxel and two dose levels of figitumumab (10 mg/Kg or 20 mg/Kg), or carboplatin paclitaxel alone. At the moment of disease progression, patients who received CT alone were treated with figitumumab with or without other CT regimen (investigator’s discretion). The addition of figitumumab to carboplatin-paclitaxel significantly increased the overall response rate (54 % versus 42 %) [33]. Exploratory analyses revealed a dose-dependent response in patients with squamous-cell histology treated with 20 mg/Kg of figitumumab plus CT with a trend towards better PFS [33].

These encouraging results lead to its subsequent evaluation in phase III trials. Two randomized phase III studies in advanced NSCLC were prematurely discontinued after Independent Data Monitoring Committees analysis indicating that the addition of figitumumab to carboplatin-paclitaxel would be unlikely to meet the primary endpoint of improving overall survival. Additionally more treatment related deaths and serious adverse events were reported in the combination arm. Cardiac toxicity was two times higher in patients treated with figitumomab (3 % versus 1.2 %). High baseline circulating IGF-1 level was predictive of better survival in figitumomab-carboplatin-paclitaxel arm [37]. Unmet primary endpoint could be attributed at least partially to the heterogeneous population of advanced NSCLC included and no available stratification according to hystological subtypes. Also, there was not an exploratory evaluation of potential biomarkers that could be eventually related to treatment response.

Finally, a third planned phase III trials evaluating the combination of figitumumab with cisplatin and gemcitabine (ADVIGO1017) was cancelled prior to study enrollment [38].

It’s somehow unfortunate that the initial encouraging activity of IGF-1R targeting combined with standard chemotherapy was not confirmed in large phase III clinical trials. Careful selection of patient population according to histological subtypes and smart trial design which could lead to the identification of putative predictive biomarkers, beyond circulating IGF-1 levels, might be more successful and permitting less costly drug approval strategies.

## IGF-1R inhibitors in sarcoma

IGF-1R axis alteration were described in many sarcoma subtypes. In Ewing sarcoma, characterized by EWS/FLI-1 translocation, enhanced IGF-1R activity has been observed. This was related to the transcriptional repression of the IGFBP-3, increasing IGF-1 ligand bioavailability with resulting IGF-1R activation. In desmoplastic small round cell tumors (DSRCT) EWS-WT1 translocation induces a threefold over-expression of IGF-1R [39, 40]. Gastrointestinal stromal tumors (GISTs) lacking of KIT and PDGFRα mutations presented significantly higher prevalence of IGF-1R amplification compared to mutated ones [31]. High IGF-1 and IGF-2 expression levels were associated to a highly malignant phenotype and negative prognostic value in wild type and mutant GISTs [41]. In addition, increased IGF-2 levels was described in many other sarcoma subtypes, such as rhabdomyosarcomas, leiomyosarcomas and synovial sarcomas, suggesting an autocrine/paracrine dependency on this pathway [41].

Based on the strong biological rational of inhibiting IGF-1R mediated signaling in sarcoma, many clinical trials were conducted or are ongoing in this patient population (Table 1).

**Table 1:** Activity of IGF-1R targeted monoclonal antibodies (moAb) in trials including sarcoma patients

Molecule	Class	Phase	Sarcoma type	No of patients	CR+PR no (%)	SD no (%)
AMG 497 (51)	Fully human IgG1 moAb	II	ESFT and DSRCT	35	2 (6 %)	17(49 %)
R1507-robatumumab, (48)	Fully human IgG1 moAb	II	ESFT	111	17 (15.3 %)	17(15.3 %)
CP-751,871- figitumumab, (50)	Fully human IgG2 moAb	I expansion cohort	advanced sarcoma (13 ESFT)	29	2 (EFST)	6 (ESFT)
						1 (SS)
						1 (FS)

*ESFT* Ewing’s sarcoma family of tumors, *DSRCT* desmoplastic small round cell tumors, *SS* synovial sarcoma, *FS* fibrosarcoma, *CR* complete response, *PR* partial response, *SD* stable disease

The most striking evidence of clinical activity emerges from Ewing sarcoma. The results of two phase II trials were recently published, evaluating the efficacy and safety of R1507 (robatumumab, a fully human IgG1 mAb to IGF-1R) in recurrent and refractory Ewing’s sarcomas and AMG 479 (fully human mAb to IGF-1R) in recurrent refractory Ewing’s family of tumors and desmoplastic small round cell tumors (DSRT). In the SARC 001 study 111 Ewing’s sarcoma patients were treated with R1507, administered intravenously at 9 mg/kg weekly. Overall response rate was 9 % (1 complete response and 9 partial responses according to RECIST criteria) and additional 21 % of patients experiencing unconfirmed partial response or disease stabilization. Thus two patterns of response were identified, 9 % of the patients achieving a robust, durable response for about 25 weeks and 6 % having short lived responses. Median progression free survival in this study was 5.7 months and overall survival 6.9 months [42].

Based on the encouraging phase I result with AMG 479 showing a complete response in one Ewing’s sarcoma patients sustained after more than 3 years and a second unconfirmed PR, a phase II trial was conducted in a population of 38 patients having a recurrent or refractory Ewing’s family of tumors (EFT) or DSRCT. Additionally a biomarker analysis was performed, exploring the relation between EWS translocation and clinical response. Two patients (one EFT and one DSCRT) achieved a partial response and almost half of overall patient population had a stable disease. Clinical benefit rate (overall response and disease stabilization for more than 24 weeks) was 17 %. PFS was about 8 weeks for EFT and 19 weeks for DSCRT. Two best responses had predominantly EWS-FLI1 type 2 transcripts, but globally no correlation could be identified between a specific EWS translocation and clinical benefit [43].

Twenty-nine patients with Ewing’s sarcoma and a heterogeneous group of other sarcoma subtypes were treated with single agent figitumumab (CP-751, 871, Pfizer, IgG2 monoclonal antibody to IGF-1R) using a dose of 20 mg/kg every 3 weeks. Although primary endpoints were safety and tolerability, preliminary data of antitumor activity were also provided. Twenty-two patients were evaluable for response and half of them presented tumor shrinkage. One Ewing’s sarcoma patients achieved a pathological complete response and one a partial response, five additional patients having some degree of tumor reduction but remaining in the category of stable disease according to RECIST criteria lasting between 4 and 16 months. Disease stabilization for 4 months or longer was also noticed in one patient having a recurrent synovial sarcoma and an additional one with fibrosarcoma [44].

A phase II single arm study of figitumumab in Ewing’s sarcoma is completing accrual with approximately 130 patients [45]. A phase II trial investigating the efficacy of SCH-717454 (robatumumab, a fully human neutralizing anti IGF-1R antibody) has planned to include 190 patients with osteosarcoma and Ewing’s sarcoma family of tumors [46]. A second trial with cixutumumab (fully human IgG1 moAb) is recruiting 185 patients in 5 arms with different sarcoma subtypes [47].

It can be concluded that monoclonal antibodies targeting IGF-1R produced some activity in sarcoma patients. The major challenge is how to select these patients and what are the best predictive biomarkers of response to these therapies.

## IGF—1R inhibitors in breast cancer

IGF-1R overexpression was observed in 44 % of breast cancer tissue specimens, showing no correlation with prognosis [48]. Circulating IGF-1 levels were associated with primary breast cancer risk. This seems to be confined to estrogen-receptor positive tumors and to be not significantly modified by IGFBP-3 levels or menopausal status [49]. High IGF-1 activation was also associated with poor prognosis in breast cancer. IGF-1 gene signature appeared to be up regulated in basal like (ER and HER2 negative) and most of the luminal-B tumors (ER positive but highly proliferative disease) [50].

There is extensive preclinical evidence supporting the synergistic growth inhibition property of combined IGF-1R and HER2 targeting treatment [18, 20, 21].

Increased IGF-1R expression was highly associated with ER status, encoded by estrogen receptor alpha (ESR1) gene. Reciprocal inhibition of ERS1 or IGF-1R transcript levels was produced by siRNA knockdown of one or the other of these targets. Furthermore it was shown *in vitro* and *in vivo* synergism of dual targeting of these pathways by fulvestrant or tamoxifen combined with h10H5, an IGF-1R monoclonal antibody [29].

Increased IGF-1R signaling has been also implicated in trastuzumab resistance. A bidirectional cross talk was detected between the two receptors in preclinical studies. Recombinant human IGFBP-3 showed significant inhibition of tumor growth in trastuzumab resistant HER2 and IGF-1R overexpressing cell lines and *in vivo* synergistic interaction with antiHER2 therapy by decreasing bioavailability of IGF-1 ligand [11, 19].

A physical interaction between IGF-1R and HER2 was found in trastuzumab resistant cells since the phosphorylation of both receptors was stimulated by IGF-1 [10]. In another study both receptors were found in an immunoprecitable complex [18]. HER2 heterodimerisation with other members of HER family is a well-known phenomenon. Besides this, heterodimers with IGF-1R were also described in trastuzumab resistant cells. Another mechanism contributing to trastuzumab resistance is p27kip down-regulation and this was stimulated by IGF-1 in some preclinical models [10].

Several phase I trials assessing the safety of IGF-1R targeted agents demonstrated clinical activity in two advanced breast cancer patients receiving AVE 1642 and one treated by AMG 479 as single agent [51, 52].

Ongoing trials in advanced breast cancer evaluate the activity of different drug combinations with IGF-1R inhibitors. Based on the fact that IGF-1 is up regulated in poor prognosis, ER positive luminal B tumors, Neo-BIG designed a neoadjuvant trial combining letrozole with MK-0646 (BIG 1–09). Unfortunately the clinical development of this protocol was temporary suspended. Additional phase II trials are evaluating the IGF-1R inhibitors associated to endocrine treatment or HER2 inhibitors in advanced breast cancer tumors (Table 2).

**Table 2:** Ongoing clinical trials with IGF-1R monoclonal antibodies (moAb) or small molecule tyrosine kinase inhibitors in association with hormonal or HER2 targeting agents

Molecule	Class	Phase	Comparison	Disease characteristics	Estimated enrollment	Trial identifier
CP-751,871 (figitumumab)	Fully human IgG2 moAb	II	exemestane +/−figitumumab	HR+ advanced breast cancer	260	NCT00372996 [53]
IMC-A12 (cixutumumab)	Fully human IgG1 moAb	II	antiestrogens +/− cixutumumab	HR+ advanced breast cancer refractory to an antiestrogen treatment	93	NCT00728949 [54]
IMC-A12 (cixutumumab)	Fully human IgG1 moAb	II	Capecitabine + lapatinib +/−IMC-A12	HER2+ previously treated with trastuzumab, an antracycline and taxane	154	NCT00684983 [55]
AMG 479	Fully human IgG1 moAb	II	exemestane or fulvestrant +/− AMG 479	HR+ advanced breast cancer	156	NCT00626106 [56]
BMS-754807	Small molecule	I/II	trastuzumab+ BMS-754807	HER2+ metastatic, failed at least one anti HER2 treatment	48	NCT00788333 [57]
MK-0646 (dalotuzumab)	Humanized IgG1	II	Letrozole +/− MK-0646	ER+ neo-adjuvant	NA	BIG 1-09

Preclinical studies suggest that mTOR inhibitors are able to up-regulate PI3K-Akt pathway by the release of the negative feedback of S6K on IRS-1 [22, 24]. Remarkable activity was seen in breast cancer patients in a phase I dose finding study of oral mTOR inhibitor ridaforolimus associated to IGF-1R monoclonal antibody, MK-0646 (dalotuzumab). Ten out of 23 patients (43 %) diagnosed with metastatic breast cancer experienced clinical activity in the expansion cohort of this study. Most of them had hormone receptor positive tumors with high proliferation rate, defined by Ki67 levels above 15 %. In this specific patient population the response rate was as high as 54 %. Based on these encouraging results a phase II study is ongoing comparing exemestane with the association of ridaforolimus and dalotozumab in HR overexpressing, HER2 negative tumors, failing 1–2 hormonal agents and maximum one chemotherapy regimen for metastatic disease [58]. Surprisingly these results were not reproduced in nine breast cancer patients included in another phase I trial combining Temsirolimus with IMC-A12 (cixitumomab, fully human IgG1 monoclonal antibody). Only one patient with breast cancer had disease stabilization in this study [59]. Overall these data are still immature and unfortunately none of these trials was designed to evaluate in parallel molecular characteristics of individual tumors that could predict eventually treatment response or resistance.

We can conclude that the main area of interest of using IGF-1R targeted agents in breast cancer is a combination strategy with endocrine treatment, HER2 and mTOR targeted agents. Clinical confirmation is in progress.

## IGF-1R inhibitors in adrenocortical carcinoma (ACC)

Metastatic and non-surgically manageable adrenocortical carcinoma (ACC) has the reputation of highly resistant disease to classical systemic therapeutic interventions. Some preclinical data suggests that IGF system has an important role in ACC pathogenesis. The vast majority of human ACC specimens display a relevant overexpression of IGF-2 transcripts compared to normal adrenal tissue. This was related to several genetic alterations such as loss of imprinting or loss of heterozygosity of the 11p15 gene locus [60, 61]. Additionally, a high overexpression of IGF-1R was found in ACC samples with concomitant downstream Akt activation, demonstrating the critical role of the pathway in the pathogenesis of this disease [61].

Preliminary antitumor activity was seen in one ACC patient treated with OSI-906 a small-molecule IGF-1R tyrosine kinase inhibitor in a phase I dose-escalation study. A randomized, double blind placebo controlled study is currently including participants evaluating the efficacy of this drug in locally advanced or metastatic ACC [62].

Fourteen ACC patients were treated in the phase I dose expansion cohort with figitumumab. The results of this study are somehow less encouraging, since no relevant clinical activity was demonstrated. However four patients experienced tumor shrinkage without meeting RECIST criteria of partial response [63].

## Conclusions and future directions

Clinical validation of IGF-1R as a target emerged with early evidence of activity, especially in Ewing’s sarcoma and other sarcoma subtypes, ACC and NSCLC. However evidence is still limited to draw a definitive and firm conclusion on which patient population may have benefit. Thus, despite the overwhelming early enthusiasm, the development of IGF-1R targeted agents arrived to an important crossroad.

**Fig. 2:**
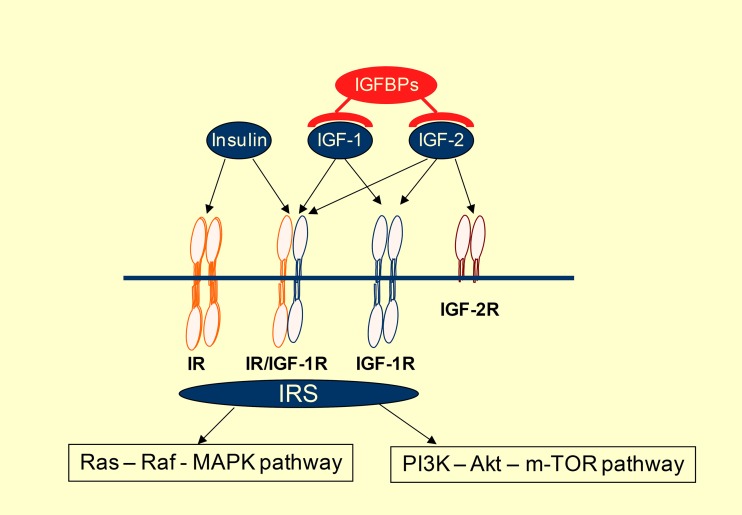
Insulin like growth factor (IGF) and insulin receptor (IR) signaling system. The availability of IGF-1 and IGF-2 ligands is highly influenced by IGFBPs (IGF-binding proteins), whereas insulin has direct access to its receptor. IGF-2 can also be sequestered by IGF-2R, which does not activate downstream signaling. Tyrosine-kinase receptors, such as holo-IR, IGF-1R/IR hybrids and holo-IGF-1R phosphorylate their adaptor proteins (IRSs), by this way conducting to downstream receptor signaling activation. Phosphoinsitide 3-kinase (PI3K)—Akt—mammailian target of rapamycin (m-TOR) is a critical pathway in IGF-1R signaling. ( IGF1, 2—insulin-like growth factor 1 and 2, IGFBPs—IGF binding proteins, IR—insulin receptor, IGF-1R—insulin-like growth factor receptor-1, IGF-2R—insulin-like growth factor receptor-2, IRS—insulin receptor substrate)

Up to now there are few, if any reliable **biomarkers** predicting response to IGF-1R targeting agents. Alterations of different IGF-1R axis components were potentially related to efficacy in preclinical studies. (Table 3) Aberrant expression of IGF-1R is detected in many cancers. Some preclinical studies conclude that IGF-1R expression is necessary but not sufficient to predict sensitivity. While phospho-IGF-1R levels seem to not correlate with in vitro effectiveness of IGF-1R targeting, total expression of IGF-1R was a better predictor of response [27, 29]. The number of IGF-1R per cell was correlated with response in some preclinical studies suggesting that somewhere between 1300 and 10000 receptors are necessary to obtain effective cell growth inhibition. These data should be interpreted with caution for many reasons. IGF-1R takes part of a complex autocrine loop involving also its ligands, IGF-1 and IGF-2, as well as intracellular adaptor proteins such as IRS-1 and IRS-2. Meanwhile ligand bioavailability is dependent on IGFBP overexpression. (Figure 2) To give an example: sarcoma cell lines resistant to BMS 536924 (small molecule IGF-1R tyrosine kinase inhibitor) expressed high levels of IGFBP-3 and IGFBP-6 whereas the sensitive ones showed high expression level of IGF-1 and IGF-2 [16]. Other investigators have reported IRS-1 and IRS-2 as predictors of response, suggesting the importance of IGF-1R axis activation in therapeutic response as well [29]. Besides IGF-1R the ligands may also activate IR and IGF-1R/IR hybrids. Generally tyrosine - kinase inhibitors are not selective for IR or IGF-1R, while monoclonal antibodies are blocking only IGF-1R and IGF-1R/IR hybrids. Thus the individual assessment of both receptors and their conformation could have an impact on response to different targeting strategies. One can also speculate that IGF-1R blockage by a therapeutic antibody leads to increased ligand availability to insulin receptor thus conducting to its compensatory activation. The role of the latter in human cancer development is frequently evoked.

**Table 3:** Potential biomarkers correlated to response to IGF-1R inhibitors in preclinical studies

	Description	Reference
IGF-1R expression level	Intensity of IGF-1R expression was correlated to in vitro response to a humanized monoclonal antibody in breast, colorectal and NSCLC cell lines. About 1,300 to 10,000 receptors per cell were necessary to achieve a meaningful cell growth inhibition.	[27, 29]
IGF-1R copy number gain	Copy number gain of IGF-1R was identified in some tumor types such as wild-type GIST, NSCLC and breast cancer. This was correlated to treatment response in NSCLC cell lines.	[25, 27, 31]
	No activating mutation if IGF-1R was identified.	
Ligands and binding proteins	IGF-1 and IGF-2 gene expression level was significantly correlated to the in vitro activity of BMS 536924 on different sarcoma cell lines, whereas IGFBP-3 and IGFBP-6 expression predicted resistance.	[16]
Receptor substrates	Either insulin receptor subsrates-1 (IRS-1) and – 2 expression was predictive of response to IGF-1R targeting agents in breast cancers. This highlights the importance of IGF-1R axis activation in therapeutic activity.	[29]
Downstream signaling	Phosphoinositide 3-kinase/Akt is a critical pathway in IGF-1R signaling. Constitutive activation of the pathway was correlated to resistance to IGF-1R targeted agents in NSCLC.	[27]

No activating mutations of IGF-1R have been described so far. On the other hand IGF-1R gene copy number gain was identified in some tumor types, such as wild-type GIST, breast cancer and NSCLC. This might support the idea that IGF-1R gene amplification and its relationship to treatment response is worthy to be evaluated.

IGF-1R phosphorylation leads to the activation of multiple signaling pathways. The antiapoptotic phosphoinositol 3-kinase (PI3K) -Akt pathway is a critical pathway in IGF-1 signaling, although it can mediate also signal from other growth factor receptors. Downstream signaling inhibition could be not only a useful pharmacodynamic biomarker, but also its examination before and after treatment could be informative regarding treatment response as well.

Taking into account the above-mentioned data, clinically quantifiable biomarkers should be developed, promoting smarter trial design to select patient population treated with IGF-1R targeted agents. Identification of molecular abnormalities of IGF-1R as well as at the level of downstream pathway components or taking IGF-1R as a part of a complex autocrine loop would be some examples from preclinical data worthy to be translated into the clinics.

Another issue that might explain, at least partly the concern regarding initial clinical results is that efficacy of IGF-1R targeting as a single agent is a rare event. Thus development of rational combinations with other anticancer agents needs to be explored. Parallel inhibition of a second growth factor receptor such as EGFR or HER2 or a concomitant downstream signaling blockade such as m-TOR or PI3K could considerably enhance antitumor activity. Combination of two HER-2 targeting antibodies directed against different epitopes of Cerb-B2 receptor showed improvement of clinical activity in the treatment of HER-2 amplified metastatic breast cancer. Pertuzumab inhibits HER-2 dimerization by preventing its pairing with other members of HER receptors. Combined treatment with trastuzumab and pertuzumab proved high efficacy in trastuzumab resistant metastatic breast cancer as well in the neo-adjuvant setting [64, 65]. Dual targeting of IGF-1R by two antibodies with distinct mechanism of action producing in the mean time an allosteric and a competitive blockage showed promising preclinical activity in one study [66, 67].

In conclusion the clinical development of IGF-1R targeted agents should be carefully reassessed while placing upfront the understanding and the identification of molecular markers predicting treatment sensitivity and resistance and the investigation of combination therapeutic strategies. In addition it is of utmost importance to optimally design clinical trials.
